# Noncovalent Sericin-Chitosan Scaffold: Physical Properties and Low Cytotoxicity Effect

**DOI:** 10.3390/ijms21030775

**Published:** 2020-01-24

**Authors:** Rungsima Chollakup, Pimporn Uttayarat, Arkadiusz Chworos, Wirasak Smitthipong

**Affiliations:** 1 Kasetsart Agricultural and Agro-Industrial Product Improvement Institute (KAPI), Kasetsart University, Chatuchak, Bangkok 10900, Thailand; aaprmc@ku.ac.th; 2 Thailand Institute of Nuclear Technology, 16 Vibravadeerungsit, Chatuchak, Bangkok 10900, Thailand; puttayar@gmail.com; 3 Centre of Molecular and Macromolecular Studies, Polish Academy of Sciences, Sienkiewicza 112, 90363 Lodz, Poland; achworos@cbmm.lodz.pl; 4 Specialized Center of Rubber and Polymer Materials in Agriculture and Industry (RPM), Department of Materials Science, Faculty of Science, Kasetsart University, Chatuchak, Bangkok 10900, Thailand; 5 Office of Natural Rubber Research Program, Thailand Science Research and Innovation (TSRI), Chatuchak, Bangkok 10900, Thailand

**Keywords:** sericin-chitosan scaffold, cytotoxicity, scanning electron microscopy (SEM), Fourier-transform infrared spectroscopy (FTIR), porosity

## Abstract

This research aims to utilize sericin, which is the waste from boiling silk cocoon, for the supramolecular scaffold preparation with chitosan. A suitable method for the self-assembled scaffold formation of sericin and chitosan at 1:1 stoichiometry is presented and the morphological and physical properties of the scaffold are studied. The effect of an alcohol/NaOH solution on the secondary structure of sericin protein within the sericin-chitosan scaffold, with adjusted pH, was investigated. Additionally, the scaffold was tested in a native phosphate buffer solution (PBS). The results show that sericin increases the porosity of scaffold while chitosan increases the rigidity. The self-assembled sericin and chitosan material is nontoxic to human cells and which can adhere and spread well on such support. For the effect of the molecular weight of chitosan (15,000 and 100,000 g/mol), the scaffold made from lower molecular weight (MW) chitosan provides a somewhat smaller porosity, but a similar swelling ratio and water uptake. On the basis of this research, sericin, which is a silk waste from the textile industry, can be utilized to produce a self-assembled scaffold with chitosan in order to increase the porosity of the scaffold. This type of scaffold is not toxic and can be used for the adhesion of fibroblast cells.

## 1. Introduction

Recently, new materials have been developed in interdisciplinary fields such as chemistry, biology, biochemistry, etc. [1,2,3,4]. These materials provide a variety of properties, especially for applications of new biomaterials. Self-assembly is one of the most important processes which governs functional biological structures in nature. Noncovalent interactions are important in the field of materials, which can imply molecular recognition, directionality, addressability, and programmability of the supramolecular character.

Material used for biomedical applications, such as a scaffold for tissue engineering, is required to be compatible with cell growth. It should be porous enough to create space for cell proliferation. The pore diameter must be suitable for the type of cell being cultured. Moreover, the scaffold has to be biocompatible, biodegradable, and non-cytotoxic. Research related to sericin-based scaffolds is under development. For example, sericin-polyvinyl alcohol scaffold has been used for fibroblast cell culturing [5,6,7]. Other polymers have been used on vascular tissue, joints and bones, or mixed with polyacrylamide to make a wound material [8] or the formation of porous skeletons of silk proteins, fibroblasts, and collagen for use as artificial cartilage [9]. Such natural polymers should not be toxic towards human cells [7,8,9].

Sericin is a glue protein of raw silk with a molecular weight distribution of 10,000 to 300,000 g/mol [10]. According to the degumming process, the raw silk is washed in hot water in order to remove the protein (sericin) from the raw silk. Then, the silk fibroin is spun to obtain silk thread that is used in the silk industry. The wastewater from the silk industry has a chemical oxygen demand (COD) of up to 6000 mg/L which is mostly composed of sericin protein [11]. Various techniques have been used to extract sericin from the wastewater and reuse it again, for example, ultrafiltration [11] and crystallization by cold ethanol [12], etc. Zhang reported that around 50,000 tons of sericin are produced globally from the wastewater of the silk industry [10]. This also would reduce the amount of wastewater discharged into public water systems. In addition, the effect of using membrane filtration and enzyme digestion to extract sericin from wastewater can reduce the COD in wastewater to 260 mg/L [13].

Chitosan is a product of deacylation of the chitin, which can be extracted from shrimp’s shells or other crustaceans. It is a linear polysaccharide composed of D-glucosamine which can be combined with polyanions. Chitosan has been used extensively in research [14,15], including medical research as an artificial skin or tissue engineering and wound dressing. This is mostly due to the antibacterial properties [16], the accelerated tissue growth [17], the biodegradability, as well as the potential to deliver small molecular weight drugs. Chitosan has also been used in research such as a DNA plasmid delivery and control release [16,18]. In order to improve the biocompatible properties of the scaffold it can be mixed with fibroin [19,20], gelatin [21], or collagen [22].

Concerning the sericin-chitosan scaffolds, there are evidences that have been reported on such material, for example, film made from silk sericin and chitosan with varying volume ratios was proposed as a potential application for wound dressing [23]. However, there is no known research on the biocompatibility of sericin-chitosan porous scaffolds towards human cells, which could be beneficial for potential applications in 3D cell culture or tissue engineering. It was interesting to investigate the supramolecular scaffold formation between sericin and chitosan using a self-assembly freeze-dry technique.

## 2. Results and Discussion

### 2.1. Physical Properties of Sericin and Chitosan

The sericin used as a raw material for preparation of the scaffold is similar to the one used previously in [20]. We confirmed that the main component of the sericin used is serine with a hydroxyl group at the side chain [12,24]. Additionally, aspartic and glutamic acids present in the structure are the polar components with negative charges. It has been previously postulated that glycine in this structure is more water soluble than fibroin [24]. The amount of negative charges of aspartic and glutamic acids in sericin were found to be 14.7% and 5.7% respectively. They were used to calculate the required stoichiometry ration of positively charge chitosan for the negative charge of sericin. The molecular weight of sericin determined by SDS-PAGE analysis, was 132,000 g/mol. This value of molecular weight was used to select the appropriate size of commercially available chitosan (from Polyscience) with a molecular weight of 15,000 g/mol and 100,000 g/mol for preparing the adequate scaffold.

### 2.2. The Effect of Solvent Treatment on the Scaffold Morphology

**Figure 1:**
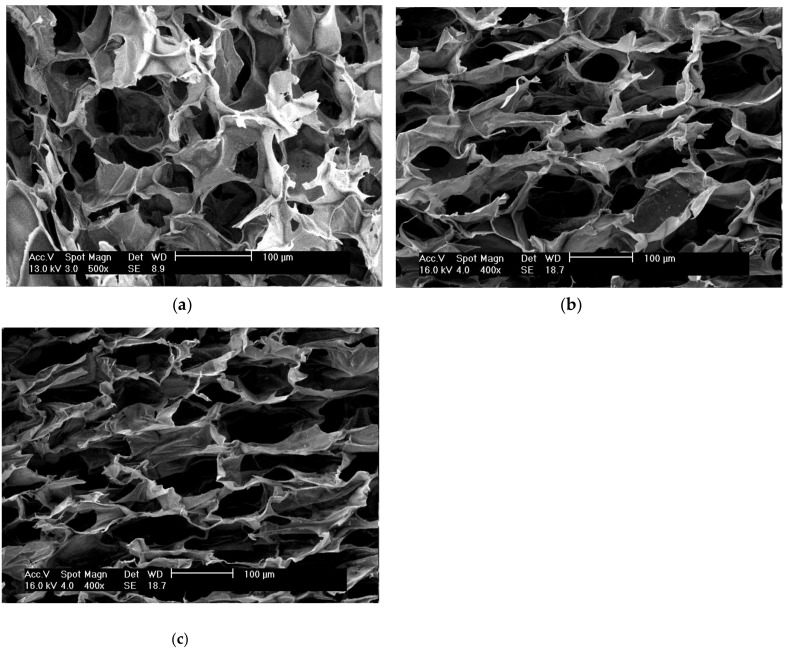
SEM images of sericin-chitosan scaffold with chitosan molecular weight (MW) of 15,000 g/mol and 1:1 stoichiometry: (**a**) Untreated scaffold A1 at X500 magnification, (**b**) methanol treated scaffold A2, and (**c**) ethanol treated scaffold A3 at X400 magnification.

To analyze the effect of solvent treatment on the sericin-chitosan scaffold, we prepared three sets of samples, namely untreated scaffold (A1), scaffold treated with the mixture of methanol/NaOH (A2), and scaffold treated with ethanol/NaOH (A3). The morphology of the material investigated with scanning electron microscopy (SEM) shows, for all samples, a relatively large pore size (Figure 1). However, the untreated sample (A1) presents symmetrical pores but scaffolds treated with methanol (A2) and ethanol (A3) present asymmetrical pores. It is worth noting that the pore size of ethanol (A3) is smaller than that of the same treatment but with methanol (A2). On the basis of the treatments with alcohol, methanol (A2) was found to have the highest porosity, whereas ethanol (A3) had a slightly reduced porosity (Table 1) due to the shrinkage of sericin under ethanol [25]. From the mechanical properties point of view, A1 was easy to break, but after treatment the scaffold with either methanol (A2) or ethanol (A3), the compressive strength of the scaffold was increased, probably because alcohol affects the structure of protein via secondary structure reformation. This suggests that alcohol could have the ability to transform the secondary structure of sericin into β-sheet and, then, induce the crystallization in sericin [20,25]. Although there were no significant differences in the compressive strength between samples A2 and A3, sample A2 was selected for further investigation due to its important porosity.

### 2.3. Effect of Molecular Weight of Chitosan on the Characteristics of the Scaffold

To further investigate the effect of the molecular weight of chitosan on the properties of the scaffold, 15,000 and 100,000 g/mol samples were used for the preparation of sericin/chitosan at 1:1 and pure chitosan. The physical properties and cell biocompatibility of scaffolds were investigated.

#### 2.3.1. Characteristic of Sericin-Chitosan Scaffold

**Figure 2:**
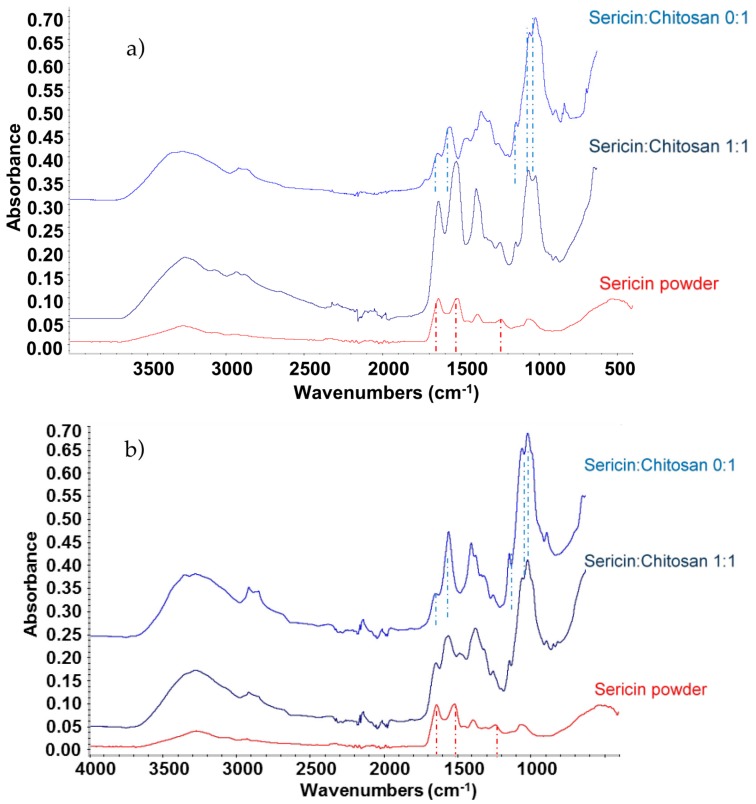
FTIR spectra at wave number during 400 to 4000 cm^−1^ of (**a**) sericin-chitosan scaffold with chitosan MW of 15,000 g/mol and (**b**) sericin-chitosan scaffold with chitosan MW of 100,000 g/mol.

The chemical composition of sericin powder, chitosan, and sericin-chitosan scaffold were analyzed with Fourier-transform infrared spectroscopy (FTIR) and data collected, as shown in Figure 2. The sericin powder presents peaks at 3280 cm^−1^ of the –OH group, at 1660 to 1670 cm^−1^ of amide I, at 1530 to 1540 cm^−1^ of amide II, and at 1240 cm^−1^ of amide III [26,27,28]. Sericin powder also presents a peak at 1400 cm^−1^ which corresponds to hydroxyl groups of serine and tyrosine [28]. For the chitosan scaffold, the main peaks are observed at 1646 and 1584 cm^−1^ which corresponds to C=O and NH_2_ of the core structure of chitosan, the peak at 1150 cm^−1^ correlated to the stretching of the C-O-C and peaks at 1059 and 1026 cm^−1^ related to the stretching of C=O [29]. For the sericin-chitosan scaffold at the ratio of 1:1 for both 15,000 and 100,000 g/mol MW, significant peaks of sericin and chitosan were found for both mixtures. The peaks of the amide group I and II, which are the arrangement of the β-sheet sericin protein in the skeletal form [30], and the peaks of C=O and NH_2_ of the chitosan molecule, were found at 1150, 1059, and 1026 cm^−1^. 

However, only for the sericin-chitosan scaffold at the ratio of 1:1 with a molecular weight (MW) of chitosan at 15,000 g/mol, the absorption band at 1561 cm^−1^ for N-H resonance of chitosan disappeared, indicating a strong interaction between the sericin and chitosan structure. These results are consistent with a previous report [31] which described the FTIR results of the silk fibroin and chitosan scaffold at a ratio of 1:1. Concerning the effect of a higher chitosan MW of 100,000 g/mol (Figure 2b), the FTIR pattern of sericin/chitosan at 1:1 presents all important peaks of chitosan at 1564, 1150, 1059, and 1026 cm^−1^ suggesting no interaction between sericin and chitosan. High viscosity of chitosan with a MW of 100,000 g/mol resulted in poor distribution of sericin and chitosan.

**Figure 3:**
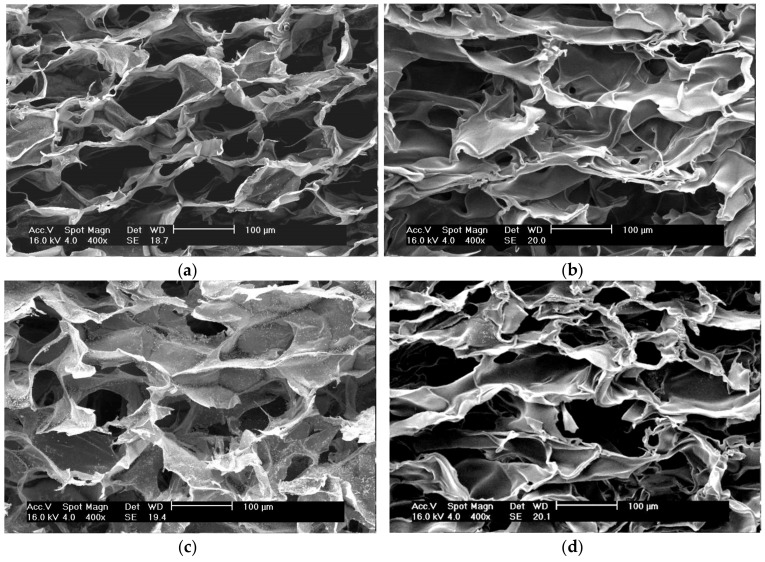
SEM images of (**a**) sericin-chitosan scaffold with chitosan MW of 15,000 g/mol with 1:1 ratio, (**b**) chitosan scaffold with a MW of 15,000 g/mol, (**c**) sericin-chitosan scaffold with chitosan MW of 100,000 g/mol at a ratio of 1:1, and (**d**) chitosan scaffold with a MW of 100,000 g/mol (at X400 magnification).

SEM images of sericin-chitosan scaffold with chitosan MW of 15,000 g/mol (Figure 3a) showed more uniform, and small symmetrical pore size as compared with the sericin-chitosan scaffold with chitosan MW of 100,000 g/mol (Figure 3c). But chitosan scaffold at 15,000 g/mol (Figure 3b) presents thicker between cells than sericin-chitosan scaffold (Figure 3a). In the case of the chitosan scaffold with chitosan MW of 100,000 g/mol (Figure 3d), its morphology appeared the same as the effect of chitosan MW of 15,000 g/mol (Figure 3b).

**Figure 4:**
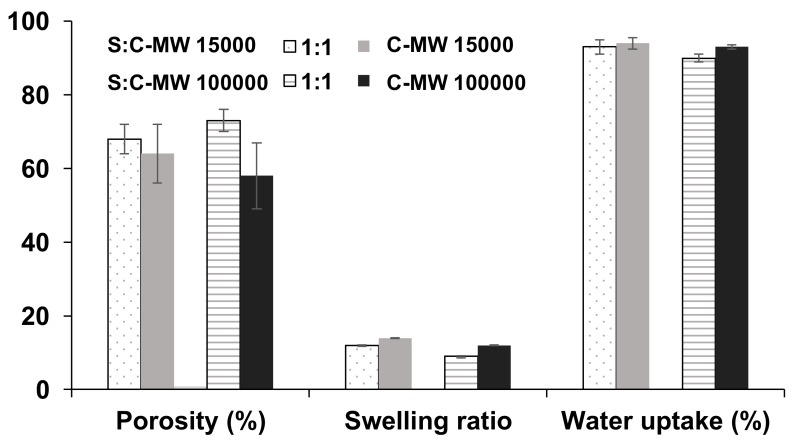
Effect of MW of chitosan on porosity, swelling ratio, and water uptake of sericin-chitosan scaffold with chitosan MW of 15,000 g/mol and 100,000 g/mol at a ratio of 1:1 and pure chitosan.

Concerning other characteristics, the porosity of the sericin-chitosan scaffold at a ratio of 1:1 seems to be less dependent on MW, however the porosity of the pure chitosan tends to be smaller than that of the sericin-chitosan scaffold (Figure 4). The swelling ratio for a low MW chitosan seems to be slightly higher than the high MW chitosan for both scaffold ratios. Because a high MW chitosan with sericin provided more network structure and yielded more porosity, there was less swelling as compared with a lower MW chitosan. This result has also been supported by previous studies [32], which found that the degree of swelling of chitosan film was higher than sericin film. However, the water uptake for all samples, sericin and chitosan and pure chitosan scaffolds, are very similar. It is worth noting that the higher viscosity of a high MW chitosan on sericin-chitosan scaffold resulted in sericin having difficulty dispersing in the chitosan solution. 

**Figure 5:**
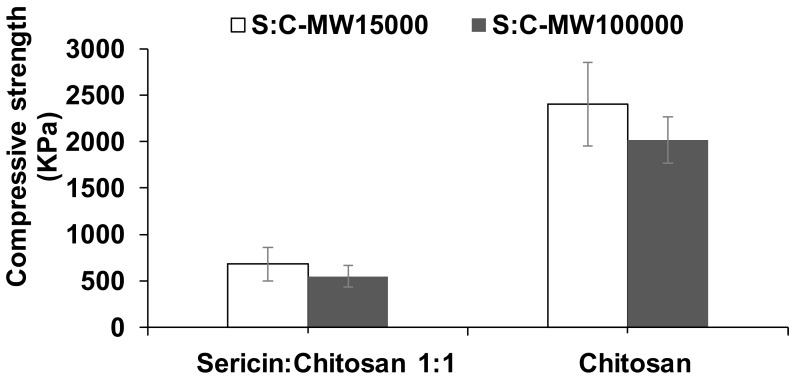
The effect of chitosan MW 15,000 g/mol and 100,000 g/mol on the compressive strength of sericin-chitosan scaffold at a ratio of 1:1 as compared with pure chitosan.

Moreover, chitosan is probably responsible for strengthening the scaffold, as demonstrated by the compressive strength results (Figure 5). The chitosan scaffold gave almost four times higher compressive strength than that of the sericin-chitosan scaffold. This is important to scaffold design for particular applications, where higher or lower compressive strength is desirable. However, chitosan scaffold with thick pore cells can absorb water and hold water in the cells slightly better than the sericin-chitosan scaffold. Generally, a scaffold should provide a mechanical strength in the range of 0.05 to 350 MPa, for example, the moduli of poly(ethylene glycol)-terephthalate scaffolds were 0.05 to 2.5 MPa [33].

**Figure 6:**
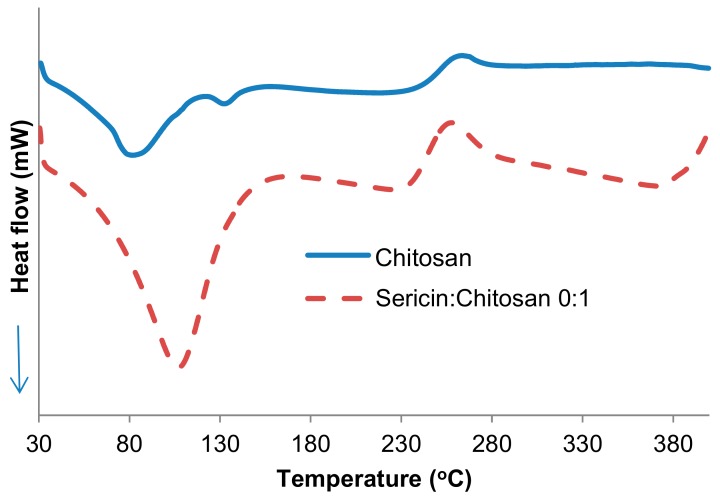
The DSC thermograms of the sericin-chitosan scaffold (chitosan MW 15,000 g/mol) at a ratio of 1:1 and pure chitosan.

The thermal behavior of the sericin-chitosan scaffold (chitosan MW 15,000 g/mol) at a ratio of 1:1 and pure chitosan was investigated by DSC measurement, as shown in Figure 6. The pure sericin could not be formed as a porous structure, and thus it was not compared. The endothermic peak of the pure chitosan at 100 °C was attributed to the evaporation of moisture within structure. The exothermic peak at 250 °C was also found due to the depolymerization of molecular structure at a high temperature with the presence of oxygen and also the decomposition of the chitosan structure [29]. In the case of sericin-chitosan scaffold at a ratio of 1:1, the endothermic peak at almost 100 °C was found and this can be explained due to a loss of water molecule in both sericin and chitosan structures. Moreover, the important endothermic peak of the sericin-chitosan scaffold at 126 °C, attributed to the movement of sericin molecules when the heat was obtained [28], and the exothermic peak at 250 °C were also found [29]. The thermal behavior of the sericin-chitosan scaffold with MW of chitosan at 100,000 g/mol gave the same tendency result.

#### 2.3.2. Cytotoxic Effect of Sericin-Chitosan Scaffolds

**Figure 7:**
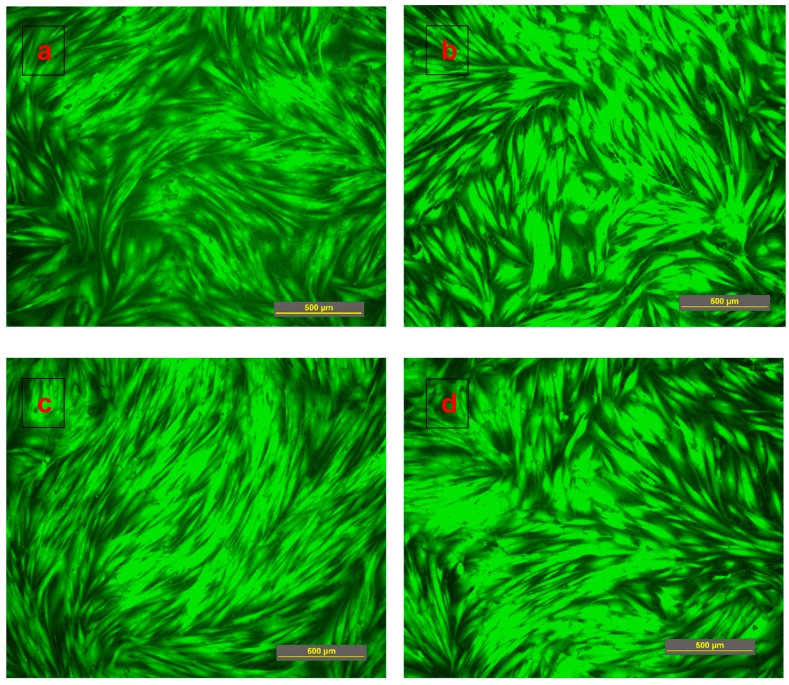
Cytotoxicity fluorescence test with human dermal fibroblasts: (**a**,**b**) Sericin-chitosan scaffold with chitosan MW of 15,000 g/mol at a ratio of 1:1 and pure chitosan and (**c**,**d**) sericin-chitosan scaffold with chitosan MW of 100,000 g/mole at a ratio of 1:1 and pure chitosan for 8 days of incubation.

**Figure 8:**
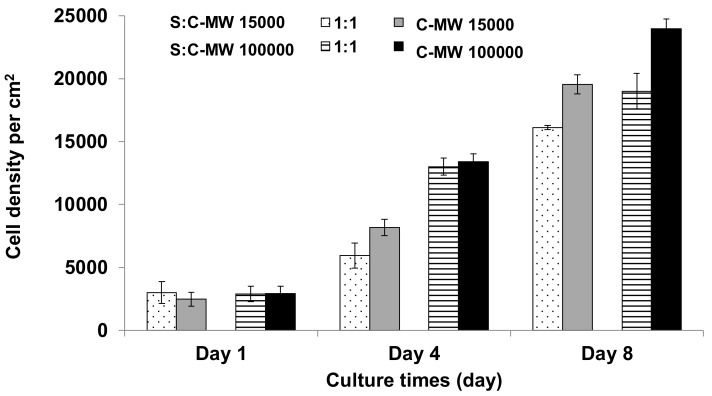
Increase cell density in the media incubated with sericin-chitosan scaffold with chitosan MW of 15,000 g/mol and 100,000 g/mol at a ratio of 1:1 as compared with pure chitosan.

The cytotoxicity study for sericin-chitosan scaffolds was done according to the procedure reported previously by [34,35]. Essentially, when samples were incubated in a cellular medium (here for three days), the soluble residuals transfer to media and, then, the media solution was used for the cell cultivation. The cells spread throughout with a long shape appearance and became a confluent monolayer with no toxicity reported for sericin-chitosan scaffolds at a ratio of 1:1 and pure chitosan for both chitosan MWs even after eight days of incubation (Figure 7). All healthy cells are fluorescent stained with no red dye visible, which would indicate the membrane perforation. Figure 8 shows cell density per area in growth media of sericin-chitosan scaffold at different culture times. We determined the cell density by counting cells from five different areas using an objective lens 10X on days one, four, and eight. Evidently, cell density increased from one to eight days. The cell density of pure chitosan at MW = 15,000 g/mol was somewhat larger than that of the sericin-chitosan scaffold. However, after four days at a higher chitosan MW, the cell density between the scaffold and pure polysaccharide was similar. This can be explained by the network of chitosan molecules that yielded to a thicker wall in scaffold pore, and therefore fibroblast cells grow more crowded than that of the lower chitosan MW. For all cytotoxicity tests, nontoxicity of this sericin-chitosan scaffold on fibroblast cell was confirmed.

## 3. Materials and Methods 

Silk cocoon waste was obtained from Shinano Kenji (Thailand) Co., Ltd., Saraburi, Thailand. Chitosan (poly-D-glucosamine) with two molecular weights 15,000 and 100,000 g/mol were purchased from Polysciences Europe GmbH, Eppelheim, Germany. Chitosan had a degree of deacetylation at 85% with an amine content of 7% to 12%. The PBS buffer (10X) was prepared using NaCl 80 g, KCl 2 g, Na_2_HPO_4_ 14.4 g, and KH_2_PO_4_ 2.4 g, dissolved in 1 L water and pH adjusted to 7.4. Sodium chloride and potassium chloride were purchased from Ajex Finechem Pty., Ltd., Auckland, New Zealand. Disodium hydrogen phosphate and potassium dihydrogen phosphate were purchased from Merck, Bangkok, Thailand. Acetic acid glacial was bought from QRec Chemical Co., Ltd., Auckland, New Zealand.

### 3.1. Scaffold Preparation

The sericin-chitosan scaffold was prepared according to a previously reported procedure [18]. Stoichiometry of sericin and chitosan was calculated to be 1:1 based on the mole value of negative groups of amino acids (aspartic acid and glutamic acid) in sericin. The procedure for the scaffold preparation is presented briefly as follows: Sericin was prepared at 5% concentration in distilled water. Samples were heated at 80 °C for 2 h. The chitosan solution at 5% concentration was prepared in 1% acetic acid. Sericin and chitosan were mixed and put into a mold (cylindrical shape, 3 cm in diameter and height). The mold was covered with parafilm and placed in a freezer at −18 °C for 24 h. Then, frozen samples were lyophilized for 24 h. Sample A1 was used without any other treatment. Samples for alcohol treatment were immersed in the mixture of alcohol and 1 N NaOH (1:1) and methanol (A2) and ethanol (A3). It is known that alcohol can alter the beta sheet structure of protein (sericin in this work) [36] and sodium hydroxide can neutralize the effect of acetic acid in a chitosan solution. Finally, the scaffold was frozen and, then, freeze-dried. Initial characterization was done for the chitosan MW of 15,000 g/mol. Then, the optimized immersing process (methanol) was selected. The effect of chitosan MW of 15,000 and 100,000 g/mol in the mixture with sericin was studied.

### 3.2. Scaffold Characterization

The pore morphology of the sericin-chitosan scaffold was examined by scanning electron microscopy (XL Series, XL30, Philips, The Netherlands) at an accelerating voltage of 10 kV. 

The functional groups of sericin powder and sericin-chitosan scaffolds at different ratios were examined by attenuated total reflection Fourier transform infrared, (Thermo Nicolet 380, Thermo Electron Corporation, Beverly, MA, USA) using a Ge crystal probe. The spectra at wave number of 4000 to 400 cm^−1^ were recorded by 64 scan summations and a 4 cm^−1^ resolution.

The porosity inside the scaffold was analyzed by hexane displacement technique [36] as hexane could permeate into the pores without swelling or shrinking the matrix (shown in Equation (1)). The scaffold (dry weight, W_1_) was immersed in a given volume (V_1_) of hexane for 5 min. The total volume of hexane and the hexane-impregnated scaffold was recorded as V_2_. The hexane-impregnated scaffold was then removed from the cylinder and weighted (W_2_). The residual hexane volume was calculated using hexane density (*p*_H_).

% Porosity = [(W_2_ − W_1_)/*p*_H_]/(V_2_ − V_1_) × 100 (1)

Swelling properties and absorption ability were determined by placing the scaffold in a hot-air oven at 65 °C for 24 h. Then, the sample was weighed and immersed in distilled water for 24 h. After that, the sample was weighed again. The swelling ratio and water uptake (absorption ability) were calculated using Equations (2) and (3) [7]:

Swelling ratio = (W_s_ − W_d_)/W_d_ (2)

Water uptake (%) = [(W_s_ − W_d_)/W_s_] × 100 (3)

where W_s_ and W_d_ are the wet weight and dry weight of the sample, respectively.

Scaffolds were analyzed for compression strength using a Universal Testing Machine (Shimadzu AGS5kN, Tokyo, Japan) with a load cell of 100 N at a strain rate of 1 mm/min until 60% reduction in sample height.

The melting temperature and heat of fusion of the scaffolds were determined using DSC (DSC1, Mettler Toledo, Toledo, OH, USA). Samples were run from 30 to 200 °C at 10 °C per min. 

### 3.3. Cytotoxicity Test of Scaffold

Sericin and chitosan at a ratio of 1:1 and pure chitosan were incubated in Dulbecco’s modification of Eagle medium (DMEM) containing 10% FBS and 1% penicilin-streptomycin for 3 days at 37 °C [33]. This media is the most broadly suitable medium for many adherent cell phenotypes and called for conditioned media (CM). We used primary cells, which were human dermal fibroblasts obtained from ATCC passages 10 to 12. Then, human dermal fibroblasts were cultured in 12-well tissue culture plate with a cell density of 20,000 cells/plate. After cell adhesive and surface dispersion on a 12-well tissue culture plate for 2 h, the specimen was incubated in CM media for 16 h at 37 °C and 5% CO_2_. Live-dead cell viability assay was used for a set of proprietary multicolor dyes that shows enhanced fluorescence upon entering live cells. Dead cells resulted in red fluorescent. This method could detect poison substances in the sample by changing the CM every day with fresh CM in the media. Then, the cell nuclei were stained using biz-Benzamide and cell numbers were counted, this experiment was carried out for 3 replicates. Moreover, cell growth on the scaffold was evaluated by coating the scaffold surface with gelatin to activate cell adhesion before cell culture. Cells did not naturally attach on the scaffold, and therefore we used gelatin to promote the initial attachment. Cells were stained by calcein AM for analysis of cell adhesion on the scaffold.

## 4. Conclusions

In this research, sericin and chitosan were studied to form a scaffold structure with variations of alcohol treatment on morphological and physical properties of scaffold. The appropriated method for forming the self-assembled scaffold between sericin and chitosan at 1:1 stoichiometry was treated with a 1:1 (v/v) methanol/NaOH solution to change the secondary structure of protein sericin and then adjust pH to neutral. The stoichiometric ratio of sericin and chitosan was at 1:1 and was compared to pure chitosan. The results show that sericin somewhat increases the porosity of the scaffold as compared with pure chitosan, while chitosan increases the rigidity. The self-assembled scaffold of sericin and chitosan is nontoxic to skin cells and the cells can adhere and spread on it. This is important to the potential application of such biomaterials for cellular growth. In the case of the molecular weight of chitosan (15,000 and 100,000 g/mol), the scaffold prepared from a lower MW chitosan provides less viscosity, which exhibited better homogeneous distribution with sericin. As it is elucidated in this research, we can utilize sericin from silk waste to produce the self-assembled scaffold by mixing with chitosan in order to enhance porosity and cell adhesion with no toxicity.

## Figures and Tables

**Table 1:** Dimension, porosity, and compressive strength of sericin-chitosan scaffold with chitosan MW of 15,000 g/mol and 1:1 stoichiometry.

	Diameter × Height (cm^2^)	Porosity (%)	Compressive Strength (kPa)
A1	2.43 × 1.01	72.68 ± 3.01	45 ± 8
A2	2.13 × 0.69	78.83 ± 1.89	652 ± 121
A3	2.10 × 0.59	67.41 ± 8.91	609 ± 66
